# Traditional complementary feeding or BLW (Baby Led Weaning) method? – A cross-sectional study of Polish infants during complementary feeding

**DOI:** 10.3389/fped.2022.992244

**Published:** 2022-10-21

**Authors:** Agnieszka Białek-Dratwa, Oskar Kowalski, Elżbieta Szczepańska

**Affiliations:** Department of Human Nutrition, Department of Dietetics, Faculty of Health Sciences in Bytom, Medical University of Silesia in Katowice, Zabrze, Poland

**Keywords:** child nutrition, baby led weaning (BLW), expanding the diet of infants, complementary feeding, child diet

## Abstract

Baby-led weaning (BLW) is an increasingly well-known method of complementary feeding for infants. The BLW method is based on the fact that the baby becomes physically ready to eat on its own and can henceforth effectively supplement its diet, which was previously based on breast milk or formula milk. The aim of the study was to compare complementary feeding among mothers using and not using the BLW method. The study took into account, among other things, the frequency of spoon feeding, eating from the family table, and eating meals with a pulpy consistency. The study also determined the frequency of the risk of choking/gagging, food regurgitation or the occurrence of vomiting during a meal in the study groups. Material and method: a cross-sectional survey was conducted among mothers of children from 6 months to 36 months of age residing throughout Poland.

**Results:**

The study group was divided into three subgroups: mothers using BLW (M-BLW), mothers not familiar with the BLW method (M-NoBLW)), mothers not using the BLW method - mothers using the spoon-feeding method) (M-TS). Among the mothers surveyed, 413 women (63.93%) used the BLW method, 222 mothers (34.36%) did not use the BLW method of which 50 (7.73%) of these were unfamiliar with the method, and 172 (26.62%) simply did not use it. Among M-TS mothers, the child was most often entirely or mostly spoon-fed by an adult (73.84%), and the same was true for the M-NoBLW group (70.0%). In the M-BLW group, 58.60% of children were half-fed by an adult with a spoon. half ate independently.

**Conclusions:**

Infants fed by the BLW method were more likely to have their diets expanded after 6 months of age, they were also more likely to be given products from the family table than children fed traditionally with a spoon. Full BLW was implemented by only 29% of children in the BLW group. The vomiting reflex, spitting food out of the mouth, and gagging, were more common among children fed by the BLW method. In contrast, choking occurred comparably often in both groups - in 5.4% of spoon-fed children and 6.9% of BLW-fed children.

## Introduction

Baby-led weaning (BLW) is an increasingly well-known method of complementary feeding for infants (1, 2). The BLW method is based on the fact that the baby becomes physically ready to eat on its own and can henceforth effectively supplement its diet, which was previously based on breast milk or modified milk (3). This process is managed by the child himself, using his skills and his instinct. Brown and Lee, in their definition of BLW, recognize this practice, in which the infant feeds itself, and feeding by the parent or giving smooth purees may occur occasionally, accounting for up to 10% of total feeding time (4). First solid foods, often referred to as complementary foods, are not intended to replace breast milk or formula milk, but should be in addition to it (1, 5).

An interesting issue in infant feeding during the expansion of the baby's diet is the mother's control over the process. Due to the development of appetite regulation, it is beneficial to feed the child according to its needs (3). The recommendations of the Polish Society for Paediatric Gastroenterology, Hepatology and Nutrition (PTGHiŻD) (6), based on the recommendations of The European Society for Paediatric Gastroenterology Hepatology and Nutrition (ESPGHAN) (5), take into account that the child should decide whether to eat food and in what quantity. Certain attitudes on the part of the parents, such as forbidding, urging, forcing, and other similar reactions, cause defensive behavior in the child and are often the cause of feeding problems. Brown and Lee (4, 7–9) noted that mothers who used BLW found it enjoyable for both them and the child to eat independently through their children. These women showed less controlling behaviour and approached the expansion of their infant's diet much more calmly than spoon-feeding mothers, other studies confirm (10).

For children, the opportunity to eat independently also has other benefits, not only related to nutrition. Rapley (11) points out that the chance to eat independently reinforces the child's sensory development and draws his or her attention to the products that are offered to him or her, rather than to the person who serves the food. Children who have the opportunity to eat freely not only improve their ability to feed themselves nutritionally, but also develop precision in grasping products and motor coordination (12). Children become active participants in the full eating process, unlike infants who do not have the chance to reach for food independently and remain inactive participants in the feeding process (10, 13, 14). Proponents of BLW suggest that this approach leads to healthier food preferences, as the infant has a greater variety of foods and role modelling by “joining in” with the family meal (15–17).

The American Academy of Pediatrics (AAP) (18), in its recommendations, recommends avoiding foods that are round and small, have a smooth and hard surface and a cross-section similar to the shape of the child's airway, therefore, when giving foods to an infant to eat alone, those that can cause choking should be avoided. Foods that are the most common cause of choking are sausages, hard candies, seeds and nuts (whole), raw apples and carrots, chewing gums, and roasted corn. The non-food items that a child is most likely to choke on are usually plastic bags and balloons, and small and round toys (19). Avoiding giving the above and similar foods to the child reduces the risk of choking. The AAP classifies eating as a circumstance conducive to choking in healthy children during movement and other activities when the child is not focusing attention on chewing (19, 18). Gagging is a vomiting reflex that allows the removal from the airway of food fragments that have entered the airway. In adults, this reflex is activated in the posterior part of the tongue, while in children it is activated closer to the anterior part of the tongue. This fact makes it easier to trigger. The reflex itself is part of the body's defense response but is rarely associated with a choking hazard. It occurs occasionally and is certainly not a danger to the child, because a piece of food is spat out before it reaches the throat (1, 20). A distinction should be made between gagging and choking. Gagging is a perfectly normal reflex that occurs in infants. Due to a lack of control over the coordination of chewing and the transfer of food to the back of the mouth, infants whoop to stop eating in the wrong way. Gagging is an important reflex and happens frequently during the introduction of solid foods when infants are developing and maturing oral motor skills as they learn to eat. Choking, on the other hand, occurs when a child's airway becomes blocked. Children may then cough in an attempt to clear the obstruction. At the same time, during choking, children may not be able to cry or cough. They may not be able to breathe, hence choking may require immediate medical attention (1, 18, 19, 20).

Despite the growing popularity of BLW and its potential benefits for child health, there is very little high-quality scientific data on this BLW method from which to conclude. Concerns about the risk of nutrient deficiencies (especially iron and zinc) for normal infant weight gain and growth (10, 21, 22), as well as the risk of gagging and choking have been pointed out by other authors (19, 18).

The aim of the present study was to test the practical application of the Baby Led Weaning feeding model among Polish mothers of children aged 6 to 36 months, including an assessment of the occurrence of differences in feeding patterns between BLW infants and infants fed traditionally with a spoon, in various aspects. Comparisons were made between the length of exclusive breastfeeding, the total length of breastfeeding, the frequency of spoon-feeding, the consistency of food consumed as complementary food, eating behaviour at the family table in both study groups. The essence of the study was to compare the risk of choking/gagging, food regurgitation or the occurrence of vomiting during a meal among children who were supplementary fed with the BLW method and those fed traditionally with a spoon.

## Material and methods

### Study group

An exploratory cross-sectional study was conducted in April 2022 among mothers of children from 6 months of age to 36 months of age (up to 3 years of age) residing throughout Poland. All participants were informed about the purpose of the study, the voluntariness of participation, and anonymity, and were asked to accept the data-sharing rules. The study was conducted among 656 women. After consideration of the inclusion and exclusion criteria, information collected from 646 women was included in the final analysis. Due to the nature of the study, approval was sought from the Bioethics Committee of the Silesian Medical University in Katowice. A decision was obtained from the relevant Bioethics Committee operating at the Silesian Medical University in Katowice to study parents’ knowledge on expanding the diet of infants (PCN/CBN/0052/KB/101/22). The Declaration of Helsinki conducted the study.

### Rationale for selecting the group

According to the current law in Poland, a mother after giving birth is entitled to take maternity leave of 20 weeks for one child, 31 weeks if she gives birth to twins, 33 weeks for three children born at the same time, 35 weeks for four children born at the same time, 37 weeks for five or more children born during one birth (23). After this time, both parents can take parental leave, which lasts 32 weeks if one child is born. However, reports from the Social Insurance Institution in Poland indicate that from January to May 2021, more than 246,000 parents, including only 1,900 men, benefited from maternity benefits for the period of parental leave (24). Therefore, mothers were invited to the study on infant diet expansion, as they are the ones who mostly stay at home with their children, spending most of their time with them, and are responsible for expanding their diet.

### Inclusion and exclusion criteria

The inclusion criteria for the study were female gender, having a child aged between 6 and 36 months (up to 3 years), consent to participate in the study, and correct and complete completion of the questionnaire. On the other hand, the exclusion criteria for the study were: lack of consent to participate in the study, incorrectly completed questionnaire, including non-response to the questions, and child's age below 6 months and above 36 months. After consideration of the inclusion and exclusion criteria, information collected from 646 women and their children was included in the final analysis.

### Research tool

The research tool was a survey questionnaire, which consisted of several parts. The data for the survey was collected anonymously using the CAWI method (Computer-Assisted Web Interview), the online survey was distributed on forums and discussion groups dedicated to mothers on Facebook, and local discussion forums, among others. As the survey was carried out using the CAWI method, the sampling was completely random (according to the assumed inclusion and exclusion criteria of the survey), however, some risk of error should be taken into account that the participants of the survey show more interest in their children's diet.

The first part of the questionnaire was a metric that asked women about their age, place of residence (rural area, city up to 20 thousand residents, city from 20 to 100 thousand residents, city from 100 to 500 thousand residents, and city over 500 thousand residents. residents) education (primary, vocational, secondary and higher), marital status (single, married, divorced, widowed, in a civil union), professional situation (maternity leave, parental leave, ½-time job, full-time job, unemployed, own business and sick leave related to another pregnancy), height and body weight. Based on the mothers' anthropometric data obtained, Body Mass Index (kg/m^2^) was calculated according to the formula, and body weight (underweight, normal weight, overweight and obese) was assessed.

The next part of the questionnaire concerned the metric data of the study children aged 6–36 months. The child's current age, current weight, and current length/height were asked. Based on the child's current age, body weight, child length/height using centile grids and 3 SD BMI for girls and boys aged 0–3 years, the WHO standard assessed children's body weight in terms of underweight, normal weight, overweight and obesity (25, 26).

In the study, mothers were asked to provide the information entered in the “Baby Health Booklet” such as the week of pregnancy in which the baby was born, birth weight, birth length, and mode of delivery (natural, planned cesarean, unplanned cesarean).

According to the position of the Polish Ministry of Health, the child's health booklet, thanks to a standardized model, contains information on the prenatal period, birth, health status after birth, patronage visits, preventive examinations, including dental examinations, history of infectious diseases, allergies and anaphylactic reactions, radiological procedures, provision of medical devices, exemption from sports activities and other information relevant to the assessment of the child's normal development from birth including measurements of weight, length/growth up to adulthood. The entries in the child's health book are made by the doctor, midwife, nurse, or another health professional immediately after the health service is provided and, where this is not possible, are completed at the next visit based on the individual internal records (27).

The next part of the questionnaire looked at how the baby was fed in the first 6 months (exclusively breastfeeding, length of breastfeeding) and how the diet was expanded (when the introduction of complementary feeding started, consistency of meals during complementary feeding -papes, pureed meals, meals ready for the baby to eat with his/her fingers; products given to the baby as complementary feeding).

The actual part of the questionnaire concerned the use of the BLW method during the expansion of the child's diet. The survey took into account whether the child was spoon-fed by an adult, whether the child ate independently, whether the carers let the child decide what the child ate and how much the child ate, the fact of using the BLW method, when the BLW method was started, whether the child was spoon-fed while using the BLW method or ate independently and to what extent.

The questionnaire was developed based on current dietary recommendations for the group of the youngest children and the method of dietary expansion developed by PTGHiŻD (6) based on ESPHGAN recommendations (5) and based on information on dietary expansion with the BLW method (1, 2, 4, 7–9, 11, 15–17, 20). In the survey, we asked mothers about the prevalence of gagging and choking among children fed traditionally with a spoon and eating according to the BLW method. Hence, in order to distinguish between these situations, they were described in the introduction, according to the definitions commonly used. A pilot study was conducted on a group of 30 mothers to validate the questionnaire and to check the relevance and acceptability of the questions included in it. Reproducibility of responses was checked by comparing responses in the same group of subjects. Pilot study II was conducted one month after the pilot study to avoid freshness effects. To assess the reproducibility of the results obtained with the questionnaire used, the parameter *ϰ* (Kappa) was calculated for each question in the questionnaire (results obtained in the pilot study and pilot study 2). For 71.2% of the questions, a very good (*ϰ* ≥ 0.80) concordance of answers were obtained, and for 22.5% of the questions, a good (0.79 ≥ *ϰ* ≥ 0.60) concordance of methods was obtained. Only 6.3% of the questions in the questionnaire analyzed had moderate (*ϰ* < 0.59) concordance between the results obtained at baseline and follow-up.

Also, the Cronbach's *α* coefficient for the standardization sample was analyzed - it was 0.86, indicating high reliability and reproducibility of the questionnaire. The pilot study allowed us to validate the questions in the questionnaire. The Cronbach's alpha coefficient for the relevant part of the survey was estimated at 0.82.

### Statistical analysis

Statistical analysis was performed using Statistica v. 13.1 software (StatSoft Inc., Tulsa, OK, United States). Statistical tests were used to analyse the variables for statistical inference. For non-parametric characteristics and bivariate tables, the *χ*^2^ test was used. The level of statistical significance adopted in the study was set at *p* ≤ 0.05.

## Results

### Characteristics of the study group of children and their mothers

Table 1 shows the anthropometric data of the study group of mothers and children. The mean age of the mothers was 30.98 ± 4.46 years. Among the children studied, the mean birth weight was 3,368.23 ± 512.38 g, length 54.18 cm ± 3.23 cm. The current age of the study children was 16.96 ± 9.1 months.

**Table 1:** Characteristics of the study group of mothers and their children (*N* = 646).

		Average	Standard deviation	Median
Characteristics of the group of mothers surveyed	Age (years)	30.98	±4.46	31
	Height (cm)	165.8	±6.05	165
	Body weight (kg)	66.86	±13.62	65
	BMI (kg/m^2^)	24.29	±2.68	23.5
Characteristics of the study group of children	Birth weight (g)	3,368.23	±512.38	3,420
	Birth length (cm)	54.18	±3.23	54
	Current age (months)	16.96	±9.1	14.5
	Current weight (kg)	10.88	±2.7	10.5
	Current length/height (cm)	82.77	±9.6	81
	Current BMI (kg/m^2^)	15.78	±2.14	15.58

Tables 2, 3 characterize the study groups of mothers and children. The mothers were most often characterized by higher education (432, 66.87%), lived in a city of 100–500,000 inhabitants (196, 30.34%), were on maternity leave (231, 35.76%), were of normal weight (368, 56.97%), and most often had one child in the family (381, 58.98%). Considering the group of children, 331 of them were girls and 315 were boys. The current body weight of the children was analyzed - age, gender, and BMI were taken into account and then these parameters were related to the BMI centile grids designed for each age group and weight normality was estimated based on them. 143 children were underweight (22.14%), 402 had normal body weight (62.23%), 46 children were overweight (7.12%) and 55 children were obese (8.51%). Most children in the study group were born naturally (335, 51.39%) and this was most often between 38 and 40 weeks of gestation (425, 65.79%). The study took into account the total length of breastfeeding and exclusive breastfeeding, which according to the WHO definition means not giving modified milk to the baby, the baby consumes only breast milk. Exclusive breastfeeding up to the age of 6 months was declared by 306 mothers (47.37%), while total breastfeeding beyond the age of 1 year was declared by 109 mothers (16.87%), but it should be taken into account that the majority of children in this study group are still breastfed, i.e., 229 children (35.45%).

**Table 2:** Characteristics of the study group of mothers (*N* = 646).

	TOTAL		BLW was not used (M-TS)		I don’t know BLW method (M-NoBLW)		BLW was used (M-BLW)	
	*n*	%	*n* 172	%	*n* 50	%	*n* 413	%
**Place of residence**								
Village	137	21.21	42	24.42	15	30.0	76	18.40
City of up to 20,000 inhabitants	39	6.04	13	7.56	5	10.0	21	5.08
City of 20–100,000 inhabitants	144	22.29	36	20.93	14	37.0	92	22.28
City of 100–500,000 inhabitants	196	30.34	52	30.23	12	24.0	128	30.99
City with more than 500,000 inhabitants	130	20.12	29	16.86	4	8.0	96	23.24
**Education**								
Basic	5	0.77	0	0	3	6.0	2	0.48
Professional	28	4.33	7	4.07	8	16.0	13	3.15
Medium	181	28.02	50	29.07	27	54.0	99	23.97
Higher	432	66.87	115	66.86	12	24.0	299	72.04
**Current professional situation**								
I don't work	96	14.86	53	30.81	5	10.0	34	8.23
Sick leave (pregnancy)	16	2.48	8	4.65	1	2.0	7	1.69
Part-time work	30	4.64	5	2.90	11	22.0	12	2.90
Full-time work	169	26.16	28	16.27	12	24.0	126	30.5
Maternity leave	231	35.76	27	15.69	17	34.0	187	45.27
Parental leave	86	13.31	44	25.58	4	8.0	36	8.71
Own business	18	2.79	7	4.06	0	0.0	11	2.66
**Mother's body weight**								
Underweight	42	6.50	12	6.98	5	10.0	23	5.57
Normal weight	368	56.97	100	58.14	25	50.0	236	57.14
Overweight	163	25.23	39	22.67	14	28.0	109	26.39
Obesity	73	11.30	21	12.21	6	12.0	45	10.90
**Number of children per family**								
One child	381	58.98	105	61.05	18	36.0	253	61.26
Two children	196	30.34	48	27.91	15	30.0	127	30.75
Three and more children	69	10.68	19	11.05	17	34.0	33	7.99

**Table 3:** Characteristics of the study group of children (*N* = 646).

	TOTAL		BLW was not used (M-TS)		I don’t know BLW method (M-NoBLW)		BLW was used (M-BLW)	
	*n*	%	*n* 172	%	*n* 50	%	*n* 413	%
**Gender of the child**								
Girl	331	51.24	98	56.98	21	42.0	209	50.61
Boy	315	48.76	74	43.02	29	58.0	204	49.39
**Current body weight according to BMI interpretation in centile grids**								
Underweight	143	22.14	40	23.26	16	32.0	87	21.70
Normal weight	402	62.23	109	63.37	28	56.0	258	62.47
Overweight	46	7.12	16	9.3	2	4.0	26	6.30
Obesity	55	8.51	7	4.07	4	8.0	42	10.17
**Type of birth**								
Natural	334	51.39	89	51.74	23	46.0	217	52.54
Planned cesarean section	141	21.83	41	23.84	15	30.0	77	18.64
Unplanned cesarean section	172	26.78	42	24.42	12	24.0	119	28.81
**Time of delivery**								
After 40 weeks	134	20.74	26	15.11	13	26.0	92	22.27
38–40 weeks	425	65.79	96	5.58	32	64.0	292	70.70
32–37 weeks	72	11.15	44	25.58	4	8.0	21	5.08
28–31 weeks	15	2.32	6	3.48	1	2.0	8	1.93
Before 27 weeks	0	0.00	0	0	0	0	0	0
**Exclusive breastfeeding**								
Less than 1 month	156	24.15	49	28.49	15	30.0	88	21.31
Up to 2 months	34	5.26	13	7.56	9	18.0	11	2.66
Up to 3 months	20	3.10	3	1.74	4	8.0	13	3.15
Up to 4 months	37	5.73	15	8.72	3	6.0	19	4.60
Up to 5 months	56	8.67	18	10.47	3	6.0	35	8.47
Up to 6 months	306	47.37	68	39.53	9	18.0	225	54.48
Does not remember	37	5.73	6	3.49	7	14.0	22	5.33
**Breastfeeding period**								
Does not remember	1	0.15	0	0.00	0	0	1	0.24
Less than 1 month	88	13.32	27	15.70	12	24.0	45	10.90
1–2 months	67	10.37	22	12.79	10	20.0	34	8.23
3–4 months	52	8.05	15	8.72	8	16.0	28	6.78
5–6 months	31	4.80	12	6.98	2	4.0	17	4.12
6–12 months	69	10.68	21	12.21	5	10.0	41	9.93
13–24 months	79	12.23	14	8.14	3	6.0	62	15.01
Over 24 months	30	4.64	2	1.16	1	2.0	27	6.45
Is still feeding	229	35.45	59	34.3	9	18.0	158	38.26

We analysed the study group of mothers and their children taking into account the use (M-TS) and non-use of the BLW method (M-BLW) and the lack of knowledge of the BLW method ((M-NoBLW) in the context of the characteristics of the study group of mothers (Table 2) and the characteristics of the study children (Table 3) and there were no significant statistical differences in terms of place of residence, education, mother's weight and in the study children in terms of gender, current weight, type of birth. There were significant differences in terms of exclusive breastfeeding and total length of breastfeeding. Mothers using the BLW method (M-BLW) breastfed significantly longer and did so more often than mothers not using the BLW method (M-TS). Mothers who did not know the BLW method (M-NoBLW) breastfed the shortest in both cases.

### Complementary feeding in the study group

The study took into account the knowledge of the BLW method and the fact of using this method for complementary feeding. The study group was divided into three subgroups: mothers using BLW (M-BLW), mothers not familiar with the BLW method (M-NoBLW)), mothers not using the BLW method - mothers using the spoon feeding method) (M-TS). Among the mothers surveyed, 413 women (63.93%) used the BLW method, 222 mothers (34.36%) did not use the BLW method of which 50 (7.73%) of these did not know the method and 172 (26.62%) simply did not use it. And 11 (1.70%) mothers could not remember whether they used or did not use the BLW method during complementary feeding (Table 4).

**Table 4:** Method of complementary feeding including the use of the BLW method in the study group of mothers and children (*N* = 646).

		BLW was not used (M-TS)		I don’t know BLW method (M-NoBLW)		BLW was used (M-BLW)		I don't remember		*p*-value
		*n* 172	%	*n* 50	%	*n* 413	%	*n* 11	%	
Start of complementary feeding	After the baby is 6 months old	82	47.67	13	26.00	263	63.68	5	45.45	*p* = 0.00000
	Between 4 and 6 months of age	85	49.4	31	62.00	143	34.62	1	9.09	
	Before the child is 4 months old	5	2.91	6	12.00	7	1.69	2	18.18	
Feeding pap during complementary feeding	Not	5	2.91	2	4.00	93	22.52	0	0.00	*p* = 0.00000
	I don't remember	0	0.00	0	0.00	3	0.73	9	81.82	
	Yes	167	97.09	48	96.00	317	76.76	3	27.27	
Feeding pap with lumps during complementary feeding	Not	59	34.30	20	40.00	97	23.49	0	0.00	*p* = 0.00820
	I don't remember	2	1.16	3	6.00	7	1.69	8	72.73	
	Yes	111	64.53	27	54.00	309	74.82	0	0.00	
Serving baby food from the family table during BLW	Sometimes	14	8.14	0	0.00	101	24.46	0	0.00	*p* = 0.0000
	Not	2	1.16	2	4.00	31	7.51	2	18.18	
	I have not used the BLW method	140	81.40	45	90.00	3	0.73	8	72.73	
	Yes	16	9.30	3	6.00	278	67.31	4	36.36	
Preparing special meals just for the baby during BLW	Sometimes	11	6.40	1	2.00	184	44.55	4	36.36	*p* = 0.0000
	Not	2	1.16	0	0.00	74	17.92	2	18.18	
	I have not used the BLW method	143	83.14	46	92.00	3	0.73	1	9.09	
	Yes	16	9.30	3	6.00	152	36.80	1	0.5%	

The initiation of complementary feeding in the M-TS group was between 4 and 6 months of age of the child (49.4%) and after 6 months of age of the child (47.67%). In the N-NoBLW group, the most common time to start complementary feeding was between 4 and 6 months of age of the child (62.0%) and in the M-BLW group after 6 months of age of the child (63.68%). The differences between the timing of the child's dietary expansion were statistically significant (*p* = 0.00000).

Feeding pap during complementary feeding was among 97.09% of children in the M-TS group, 96.0% of children in the M-NoBLW group and 76.76% of children in the M-BLW group (*p* = 0.00000). In contrast, feeding pap with lumps during complementary feeding was among 64.53% of children in the M-TS group, 54.0% of children in the M-NoBLW group and 74.82% of children in the M-BLW group (*p* = 0.00820). Considering giving the child food from the family table to eat, 9.30% of mothers in the M-TS group gave such meals to their children, 6.0% in the M-NoBLW group, while in the M-BLW group 67.31% gave such meals to their children during complementary feeding (*p* = 0.00000).

The child's independent eating before 1 year of age was allowed by 88.1% of mothers from M-BLW, 45.35% from M-TS and 36.0 from M-NoBLW (*p* = 0.00000). The child's independent decision of what to eat was 65.62% in the M-BLW group, 32.0% of M-NoBLW and 22.09 of M-TS (*p* = 0.00000) …. In contrast, M-BLW mothers in 93.22%), 84.88% from M-TS and 74.0% from the M-NoBLW group agreed with their child deciding independently how much to eat (Table 5).

**Table 5:** Eating independently, taking into account the use of the BLW method in the surveyed group of mothers and children (*N* = 646).

		BLW was not used (M-TS)		I don’t know BLW method (M-NoBLW)		BLW was used (M-BLW)		I don't remember		*p*-value
		*n* 172	%	*n* 50	%	*n* 413	%	*n* 11	%	
Eating on their own before the age of one	Sometimes	64	37.21	16	32.00	42	10.17	1	9.09	*p* = 0.0000
	Not	30	17.44	16	32.00	7	1.69	9	81.82	
	Yes	78	45.35	18	36.00	364	88.14	5	45.45	
The child decides for himself WHAT TO EAT	Sometimes	61	35.47	18	36.00	110	26.63	2	18.18	*p* = 0.0000
	Not	73	42.44	16	32.00	32	7.75	4	36.36	
	Yes	38	22.09	16	32.00	271	65.62	3	27.27	
The child decides for himself/herself how much to eat	Sometimes	18	10.47	9	18.00	21	5.08	0	0.00	*p* = 0.00007
	Not	8	4.65	4	8.00	7	1.69	8	72.73	
	Yes	146	84.88	37	74.00	385	93.22	1	9.09	

Spoon-feeding during complementary feeding was included in the study. Among mothers with M-TS, the child was most often fully or mostly spoon-fed by an adult (73.84%), and the same was true in the M-NoBLW group (70.0%). In the M-BLW group, 58.60% of the children were half fed by an adult with a spoon. half ate independently. During BLW supplementary feeding in the M-BLW group, only 29.06% of the children completely or mostly ate independently, 64.64% of the children were half fed by an adult or half ate independently (Table 6).

**Table 6:** Feeding the child with a spoon during complementary feeding and when using the BLW method (*N* = 646).

		BLW was not used (M-TS)	I don’t know BLW method (M-NoBLW)	BLW was used (M-BLW)	I don't remember	*p*-value
		*n* = 172 %	*n* = 50 %	*n* = 413 %	*n* = 11 %	
Spoon-feeding during complementary feeding	The child ate entirely or mostly on his/her own	0	0	87 21.07%	6 54.55%	*p* = 0.0000
	Baby fully or mostly spoon-fed by an adult	127 73.84%	35 70.00%	84 20.34%	5 45.45%	
	Child half fed by an adult with a spoon. half ate independently	45 26.16%	15 30.00%	242 58.60%	1 9.09%	
Feeding your baby with a spoon while using the BLW method	The child eats completely or mostly independently	0	0	120 29.06%	1 9.09%	*p* = 0.0000
	Child fully or mostly fed by an adult	14 8.14%	4 8.00%	26 6.30%	7 63.64%	
	Child half fed by an adult. half eats independently	19 11.05%	0	267 64.64%	2 18.18%	
	I have not used the blw method	139 80.81%	46 92.00%	0 0.0%	1 9.09%	

The aspect of incidents such as the child's vomiting reflex while eating, spitting food out of the mouth, gagging, choking and choking with the need for medical intervention was also examined. Among the children studied, the vomiting reflex was more frequent in children fed by the BLW method (34.90%). Spitting out food was more frequently observed among children fed with the BLW method. Gagging was more frequent among children fed with the BLW method (51.9%). In contrast, choking was observed in 5.42% of spoon-fed children and 6.94% of children fed with the BLW method. Choking requiring medical intervention was declared by 0.31% of mothers of spoon-fed children and 0.45% of mothers of children fed with the BLW method (Table 7).

**Table 7:** Comparison of incidents during spoon-feeding vs BLW eating (*N* = 646) - sum of responses is greater than 100%.

	Spoon-feeding		Feeding using the BLW method		*p*-value	V Cramer
	*N* = 646	%	*N* = 447	%		
Had a vomiting reflex	163	25.23%	156	34.90%	*p* = 0.0000	0.7486362
Spat food out of its mouth	335	51.86%	278	62.19%	*p* = 0.0000	0.7579381
Gagging	188	29.10%	232	51.90%	*p* = 0.0000	0.7312949
Choking	35	5.42%	31	6.94%	*p* = 0.0000	0.7125987
Choked and needed medical attention	2	0.31%	2	0.45%	*p* = 0.0000	0.7906299

## Discussion

In our study, we focused on the use of the BLW method during complementary feeding. We took into account whether the child was spoon-fed by an adult or whether the carers let the child decide what and how much the child would eat, the use of the BLW method, when the BLW method was started, whether the child was spoon-fed during the BLW method or ate independently and to what extent. We also assessed the consistency of meals during complementary feeding - paps, pureed meals with lumps, and meals ready for the child to eat with fingers. We also analyzed the incidence of eating-related incidents among infants, i.e., the vomiting reflex, spitting food out of the mouth, gagging, and choking in terms of spoon-feeding and the BLW method.

In recent years, there have been an increasing number of scientific reports on the BLW method. However, despite the benefits associated with this method, health professionals are reluctant to advise the adoption of this new approach, especially given the many concerns related to the possible negative impact on the child's health, increased risk of choking, and higher probability of low intake of energy and micronutrients, especially iron, because it is the child who decides the quantity and quality of the food, choosing among the different options given to him or her during meals (28–31). Although, in a 2022 Neves FS (32) survey of 458 Brazilian health professionals, the majority of respondents declared knowledge of the BLW method (82.1%). Considering the recommendation of this method in clinical practice, 38.3% of respondents indicated that they sometimes recommended it, 37.5% often and 20.5% always. This is also confirmed by the studies of D'Andrea et al. (33), conducted among 33 Canadian health professionals (lactation consultants, nurses, physiotherapists, doctors, dieticians, and occupational therapists) and Rubio et al. (34), with 579 Spanish pediatricians found that 81.8 and 79.4%, respectively, were familiar with the BLW method. The BLW method is becoming more and more popular every year. It began to gain notoriety in 2008, with the publication of Rapley and Murkett's paper entitled “Weaning your baby: helping your baby love good food.” In December 2016, a Google search (www.google.com) for nomenclature related to baby-led weaning yielded almost one million results; in November 2019, the same search reached almost nine million results. Topics related to the BLW method are very popular on online forums, blogs, social media, and supplementary feeding websites (35). Therefore, attitudes towards this method among health professionals are changing year by year.

A subsection on complementary feeding using the BLW method is included in the current PTGHiŻD) (6) recommendations based on ESPHGAN (5), which also highlights the increasing popularity of this method. The introduction of complementary foods by the (BLW) method is a child-controlled feeding method. It is based on bypassing the spoon-feeding stage by the carers and giving pulpy foods (purees, purées). When the baby can sit up unaided (around 6–7 months of age), a variety of solid foods are given in a form that is easy for him to grasp with his hand. Some authors suggest the use of the BLW method as a standard for complementary feeding, as self-awareness of satiety and appetite contributes to healthy eating and behavioral patterns in the future (4). However, contrary to previous hopes, BLW/BLISS methods do not reduce the risk of obesity (36).

In our study, children in the M-BLW group ate more often lump pulps during complementary feeding than children in the M-TS and M-NoBLW groups, which is unfortunately not in line with the BLW method.

One important point about the BLW method is that there is no definition and different models of BLW are often used, i.e., full BLW, partial BLW, and unconscious BLW. In its purest form, the BLW method should not involve spoon-feeding and the child should put food in his or her mouth independently (37). Studies by Cameron et al. also considered that children fed using the BLW method are not spoon-fed at all, but feed themselves by eating food with their hands (38). However, in many definitions of BLW, the method occurs when the proportion of purees fed and spoon-feeding per day is less than 10% of the total food (BLW = 10% or less) (4). The definition, however, is quite problematic for breastfeeding parents, especially mothers, given the subjective evaluation of feeding by the mothers surveyed, the evaluation of portion sizes, and also the play with food that takes place when using the BLW method.

Considering the principles of the BLW method, children should decide what they eat. In our study, as many as 32 mothers from the M-BLW group (7.75%) did not allow their children to make such decisions, and 110 (26.63%) allowed their children to decide sometimes. M-BLW mothers more often allowed their children to decide how much to eat, but it should be emphasized that among mothers of children fed with the BLW method some, unfortunately, did not decide for themselves. At the same time, some of the children, despite the declared BLW method of dietary expansion, were not fed according to all the principles of this method. On the other hand, it should be emphasized that only a part of mothers 11.05% half-fed their child or allowed it to eat independently.

In a study (22) among Chilean children (*N* = 261) who were fed using the BLW method - 12.2% of children were always spoon-fed, often (at least once a day) also 12.2%, sometimes 47.2% and never 28.4%.

Unfortunately, parents both in our study and in other cited studies do not always understand the use of the BLW method. Despite declaring the use of the BLW method, children were spoon-fed. For example, a cross-sectional study in Spain (*n* = 6,355 mothers) showed that the full BLW method was used in only 2.1% of an overall BLW prevalence estimated at 14.0% (39). The low adherence to BLW may be related to the lack of a standardized definition of this approach and the very individual interpretation by parents of the use of this method.

An important issue during complementary feeding is the concern among parents about gagging, and choking. Gagging is perfectly normal and occurs regularly as a reflex. Infants have no control over the coordination of chewing and moving food to the back of the mouth to swallow, so they gurgle to stop eating in the wrong way. Choking is an important reflex and occurs frequently during the introduction of solid foods as infants develop and mature oral motor skills as they learn to eat. In contrast, choking occurs when a child's airway becomes blocked. Babies may then cough in an attempt to clear the obstruction, or they may not be able to cry, cough, or make any noise at all. They may not be able to breathe, hence choking may require immediate medical attention (1, 18, 19, 20).

In our study, a distinction was made between gagging, choking, and choking in which medical intervention was needed.

Choking occurred in 5.42% of children fed with a spoon and in 6.94% of children fed with the BLW method. However, gagging occurred much more frequently than choking, i.e., in the group of children fed with a spoon 29.10% (188) had at least one gagging incident, while in the group fed with the BLW method it was as high as 51.90% (232 children).

The D'Auria meta-analysis (37) analyzed, among other things, the risk of choking during the BLW method, which can occur in infants when learning to eat independently. At the time of initiation of complementary feeding (i.e., around 6 months of age), the child may not yet have developed the oral motor skills required for safe ingestion of whole foods (such as chewing and swallowing) (30, 40). It is important to note that not all children are ready to start feeding solids at 6 months of age and earlier (41). Townsend E.'s study showed no difference in choking rates between BLW groups and children fed traditionally with a spoon (42). In contrast, a study by Cameron SL found that 30% of children fed BLW had at least one choking episode after eating solid food (38). Brown obtained similar results in an observational study in a group of 1,151 infants on the risk of choking and whooping. The results of the study showed that at least one choking episode (choking) occurred in 11.9% in the BLW-fed group, in 15.5% in the so-called “loose BLW” group, and 11.6% in the traditional spoon-feeding group, with no significant differences between groups (43). In a study by Quintiliano-Scarpelli D et al. the incidence of gagging, choking, and Suffocation was very high at 78.2%, 28.4%, and 3.1%, respectively (23). In our study, incidents of choking were significantly less than in other studies. In contrast, the vomiting reflex, spitting food out of the mouth, and gagging were more frequent among children fed using the BLW method than with traditional spoon-feeding.

A study by Addessi E. et al. (44) discussed whether the BLW method was associated with developmental milestones such as unsupported sitting, crawling and uttering first words. Their study showed that BLW approaches were associated with advanced motor milestones, but none of the feeding variables were associated with the age at which infants spoke their first words. However, a causal relationship between BLW and motor development cannot be established from these studies, but it cannot be excluded that infants' interactions with food for a few weeks may provide them with experiences that can influence cognitive and motor development (44). Further research especially follow up is needed to assess how the BLW method affects psychomotor development in children over several years.

## Strengths and limitations of the study

The results of our survey must be interpreted by taking into account its limitations. A limitation of the study is the lack of differentiation of the study group in terms of education level (mainly higher education). All information was provided by the mothers, which may cause information bias. Our study was a retrospective study, which may influence the occurrence of a false memory effect, especially in the group of mothers of older children aged 2–3 years, regarding the details of the expansion of the infants' diet.

In addition, the survey was conducted using the CAWI method, which is repeatedly criticized for its lack of insight into the data collection process, although it is worth noting that this type of data collection method is widely accepted and convenient for collecting large amounts of information in groups that are often difficult to access.

An advantage of the study is the size of the group of 646 mothers; until now, most studies on the application of the BLW method have been conducted in smaller groups. It is also worth mentioning at this point that very few studies have been conducted on this topic so far, especially in Poland.

## Conclusions

Infants fed using the BLW method were more likely to have their diet expanded after 6 months of age, and were also more likely to be given products from the family table than children fed in the traditional way using a spoon. Some children fed with the BLW method were not fed according to all the principles of the method. Full BLW was implemented by only 29% of the children in the BLW group, and 64% of the children in the BLW group were half-fed using a spoon. The vomiting reflex, spitting food out of the mouth, and gagging, were more frequent among children fed with the BLW method. On the other hand, choking occurred comparably often in both groups - in 5.4% of children fed with a spoon and 6.9% of children fed with the BLW method. Mothers using the BLW method (M-BLW) breastfed significantly longer and did so more often than mothers not using the BLW method (M-TS). Mothers who did not know the BLW method (M-NoBLW) breastfed the shortest in both cases.

## Data Availability

The raw data supporting the conclusions of this article will be made available by the authors, without undue reservation.
